# Evaluation of Exfoliated Graphite to Graphene in Polyamide 66 Using Novel High Shear Elongational Flow

**DOI:** 10.3390/polym10121399

**Published:** 2018-12-17

**Authors:** Justin W. Hendrix, Ryan Szeto, Thomas Nosker, Jennifer Lynch-Branzoi, Thomas J. Emge

**Affiliations:** 1 Department of Chemical and Biochemical Engineering, Rutgers University, 607 Taylor Road, Piscataway, NJ 08854, USA; 2 Department of Materials Science and Engineering, Rutgers University, 607 Taylor Road, Piscataway, NJ 08854, USA; ryanxszeto@yahoo.com (R.S.); jklynch@soe.rutgers.edu (J.L.-B); 3 Department of Chemistry and Chemical Biology, Rutgers University, 610 Taylor Road, Piscataway, NJ 08854, USA; emge@chem.rutgers.edu

**Keywords:** graphene, graphene polymer matrix composite, polyamide 66, elongational flow, hydrogen bond

## Abstract

Graphene has been publicized as the game changing material of this millennium. To this day, scalable production leading to exceptional material properties has been difficult to attain. Most methods require harsh chemicals, which result in destroying the graphene surface. A method was developed, exploiting high speed elongational flow in a novel designed batch mixer; creating a distribution of pristine few to many layer graphene flakes. The method focuses on exfoliating in a molten polyamide 66 (PA66) matrix, creating a graphene reinforced polymer matrix composite (G-PMC). The process revealed that high speed elongational flow was able to create few layer graphene. Graphite exfoliation was found driven in part by diffusion, leading to intercalation of PA66 in graphite. The intercalated structure lead to increases in the hydrogen bonding domain, creating anisotropic crystal domains. The thermal stability of the G-PMC was found to be dependent to the degree of exfoliation, PA66 crystal structure and composite morphology. The aim of this research is to characterize uniquely produced graphene containing polymer matrix composites using a newly created elongational flow field. Using elongational flow, graphite will be directly exfoliated into graphene within a molten polymer.

## 1. Introduction

Graphene has been publicized as the leading edge material of this millennia. In the past two decades, the 2-D material has been considered for use in applications, such as thermal management [1], environmental remediation and filtration [2,3], biotechnology [4,5] and lightweight, structural materials [6]; due to its exceptional mechanical properties [7]. Many exfoliation methods have been developed to separate graphene from graphite and produce un-oxidized graphene flakes, including electrochemical [8,9], chemical oxidation and expansion [10,11] and liquid phase [12,13]. However, these approaches are costly, multistep methods that use or produce harmful byproducts. Since the graphene is often extracted from a sacrificial medium, defects are often introduced on the graphene surfaces and edges, which may reduce some properties [14,15]. The challenge is finding a viable, inexpensive, less complex process that permits property retention.

An alternative approach would be to cultivate an exfoliation method, for which the matrix is not a sacrificial phase but rather becomes part of the end use application, for example, a graphene contained reinforced polymer nanocomposite. In order to do that, the matrix must impart significant shear forces to exfoliate flake graphite to graphene nanoflakes. Thus, it is important to study scalable polymer processing techniques that are optimized for efficient graphite exfoliation.

Direct exfoliation has been known to providing an economical, top-down approach in converting the graphite to graphene [3]. The processes that provide the most advantage involve traditional plastic processing utilizing extensional flow. Using elongational flow to exfoliate graphite has been attempted but inefficient exfoliation was achieved [16]. Traditionally, current mixing technologies are not independently able to efficiently create graphene without the addition of another step that causes exfoliation. Utilizing elongational flow with the addition of an exfoliation aid, has been the direction for research in this field. Ellingham et al. [17] has shown that using twin screw extrusion and the addition of supercritical CO_2_, exfoliation was further improved through bubble formation. The bubbles were able to generate an additional extension mechanism, upon expansion of CO_2_ to further exfoliate the graphite. Those that are specifically designed for elongational flow start with pre-expanded graphite, attempting to reduce the size of graphite with inherent defects [18]. There are mixers that exploit extensional flow without the addition of an additive exfoliating component. Work conducted by Oxfall et al. [16] uses dual piston driven operation injected through a capillary to cause elongational flow mixing. A drawback is the creation of hydrostatic pressure, causing intermittent stages of no mixing. Oxall’s work disproportionally hinders efficient exfoliation. Supported in our work, we’re able to create a succession of shear strain events without any chemical aid or pretreatment. A succession of shearing events in Nosker et al. [19] produces continuous mixing, making our process an economical advantage.

Some of the earliest experimental and theoretical work in fluid flow of two immiscible liquids was pioneered by Taylor in his experiments with droplet suspension breakup in a dilute medium of dissimilar viscosity [20]. Taylor designed two methods to observe droplet deformation contributing to a difference in mechanism of deformation. The first consisted of two counter moving surfaces, creating planar shear flow. From this first experimental setup at maximum roller speed, the droplet deformed to a spheroid that distorted without breaking. The second experiment conducted was of a four roll apparatus producing planar elongational flow under the same matrix conditions. What was observed, was droplet extension occurred parallel to the direction of deformation. The extension lead to droplet rupture and breakage.

Taylor found the mechanism for breakage of droplets to be related to apparent differences in kinematic viscosity ratio defined by *p* to a droplets critical deformability; where μ_D_ is the viscosity of the dispersed phase and μ_C_, the viscosity of the continuous phase (1).

(1) $$p=\frac{\mu_{D}}{\mu_{C}}$$

The critical deformability was found to work but only up to a maximum viscous ratio of 4. Similar results were confirmed by Rumscheidt and Mason of this critical viscosity ratio to deformation [21]; suggesting that beyond this critical viscosity exist no deformation and breakage of droplets. An expansion of work by Taylor & Rumscheidt from Grace produced a slightly different view on the viscous ratio. For droplet deformation leading to breakage, Grace found that a specific deformation rate above a critical capillary value Ca_crit_ was needed; where Ca_crit_ < Ca [22]. The capillary number Ca represented the ratio of the viscous force of the matrix to the restorative force of the dispersed phase to deformation or shear; where μ is the kinematic viscosity, γ is the shear rate, σ interfacial tension and R_d_ the droplet radius (2).

(2) $$\mathrm{Ca}=\frac{{\mu R}_{d}\dot{\gamma }}{\sigma }$$

**Figure 1:**
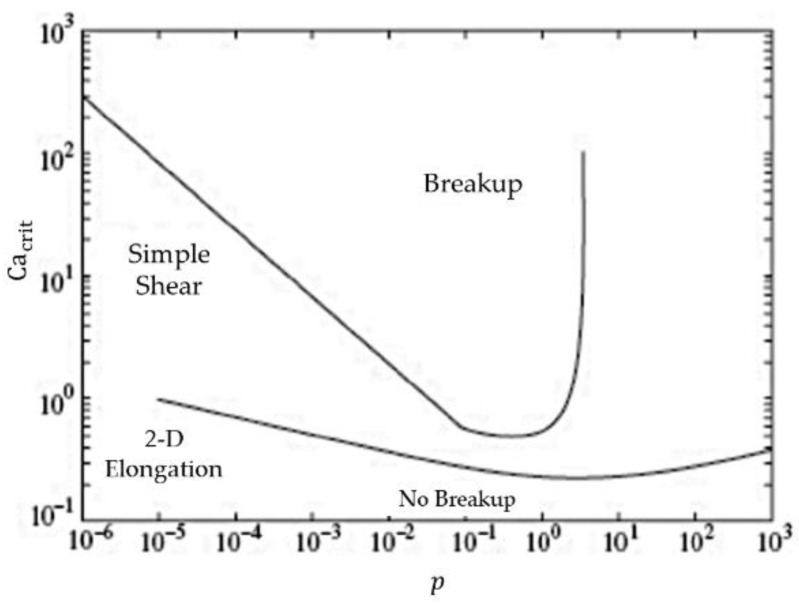
Capillary number Ca vs. viscous ratio *p* [23].

Associating elongational flow to simple shear flow figure, Grace found the critical capillary number to be smaller for the case of elongational flow. Leaving for simple shear, the critical capillary number for breakage approaching a near infinite value as the viscous ratio exceeds *p* > 1 in Figure 1 [23]. This forms the bases of 2D elongational flow as being essential for particulate breakup and deformation, based on the idea of it being seemingly independent of viscosity. This viscous independence suggests that any material system, that undergoes deformation by the application of stress, has the potential to create new surfaces. These systems include liquid-liquid in Graces work and solid-liquid systems. Particularly for solids that deform by shear stress under an inherent slip plane, like graphite.

The aim of this research is to characterize uniquely produced graphene containing polymer matrix composites using a newly created elongational flow field. Using elongational flow, the drive is to exfoliate graphite into graphene directly within a molten polymer. This work is an expansion of a method to create graphene reinforced polymer matrix composite by Nosker et al. [19]. In using a continuous polymer phase and a dispersive graphite phase, the previous art exploits fluid mechanics of droplet breakup by exceeding Ca crit across large viscous regimes created by continuous exfoliation. By exfoliating in-situ the polymer phase seeks to exceed the resistive force to exfoliation and detail a scalable method of creating a polymer matrix composites, where graphene is used as a reinforcing phase. The process may show that this method has subsequent improved particle-matrix interaction between newly formed nanographite and graphene surfaces; which is not found in traditional plastic-graphene composites. 

In this work, we use elongational flow to exfoliate 35 wt % mined graphite within polyamide 66 (PA66) to create graphene containing polyamide 66 nanocomposites (G-PA66). The components are subject to 10, 20, 30 and 45 min of elongational flow mixing. The mixed components are then characterized for their changes in structure, morphology and thermal properties. Using this single step approach to convert graphite directly to nanographite and graphene, it is suggested that a molten elongational phase can apply unique hydrodynamics on the graphite flake to produce exfoliated few, many layer graphene and nanographite at shear strain rates exceeding 10^3^ s^−1^.

## 2. Materials and Methods 

Natural flake graphite (Asbury Carbons, mills grade 3627 with 99.2% purity, diameter = 250 µm, ρ = 2.26 g/cm^3^, Asbury Carbons, Asbury, NJ, USA) was used as the exfoliating species. Polyamide 66 (PA66, Zytel 101 NC010, *T*_m_ = 262 °C, *T*_g_ = 60 °C, ρ = 1.14 g/cm^3^, DuPont, Wilmington, DE, USA) was used as the high temperature polymer in this study. Prior to processing, both components were dried in an oven to eliminate water. PA66 was dried at 85 °C under vacuum and graphite was dried at 350 °C for 4 h.

PA66, 20 wt % and 35 wt % flake mineral graphite were mixed using a high shear batch mixer imparting elongational flow. The blends were mixed at 276 °C and a shear strain rate of 2876 s^−1^, under an Ar gas atmosphere to reduce polymer degradation. 35 wt % graphite and in PA66 and PA66 were mixed for 10, 20, 30 and 45 min to produce G-PA66 samples and PA66 control samples at each mixing time Table 1, PA66 and G-PA66 extrudate (approximate diameter of 2.94 mm) was prepared for subsequent characterization.

Wide Angle X-ray Diffraction (WAXD) was performed using a Philips X-Pert Powder X-ray Diffractometer (PANalytical, Almelo, Netherlands) with a Cu (λ = 1.54 Å) K-alpha source at 45 kV/40 mA and scanned from 4° to 70° 2ϴ. The samples were scanned with a step size of 0.02° 2ϴ to detect graphitic and nylon characteristic peaks. Extrudates were notched and cryogenically fractured to produce a pristine surface. 

Thermal properties of PA66 and G-PA66 samples were determined using a Q1000 Differential Scanning Calorimeter (TA instruments, New Castle, DE, USA). PA66 and G-PA66 samples weighing 10 mg, were sectioned from the extrudate and subject to a heat, cool, reheat method at 10 °C/min from 0–300 °C under a nitrogen atmosphere. The effect of processing on the melting (*T*_m_), crystallization (*T*_c_) and glass transition (*T*_g_) temperatures of the PA66 component was determined, to include enthalpy of crystallization ΔH_c_, enthalpy of melting ΔH_m_ and % crystallinity in PA66. 

Morphology of G-PA66 samples was investigated using a Sigma Field Emission Scanning Electron Microscope (SEM) (Zeiss, Oberkochen, Germany) with Oxford EDS.

In order to better investigate the nanoflakes produced during this in situ exfoliation method, G-PA66 nanocomposites were granulated using a Spex 6700 Freezer Mill (SPEX Sample Prep, Metuchen, NJ, USA) to cryogenically impact samples and form a fine powder for viewing using the SEM. The fine powder was ultra-sonicated in isopropyl alcohol (IPA) for 5 min, then the suspension was drop coated on to a lacey carbon grid for transmission and characterization in a JEOL 2010F Transmission Electron Microscope (JEOL, Tokyo, Japan) operating at 200 kV for High Resolution Transmission Electron Microscopy (HR-TEM).

## 3. Results and Discussion

### 3.1. Wide Angle X-ray Diffraction

In our assessment of the crystalline characteristics of the X-ray diffraction results, the Scherrer Equation (3) was used to calculate the crystalline domain size of the phases in PA66, G-PA66 and Graphite. *K* = 0.9 represents the shape factor, *λ* is the incident radiation wavelength, *β* is the line broadening at half the maximum intensity of the detectable peak and ϴ is the Bragg angle in degrees.

(3) $$L=\frac{K\lambda }{\beta cosϴ}$$

**Figure 2:**
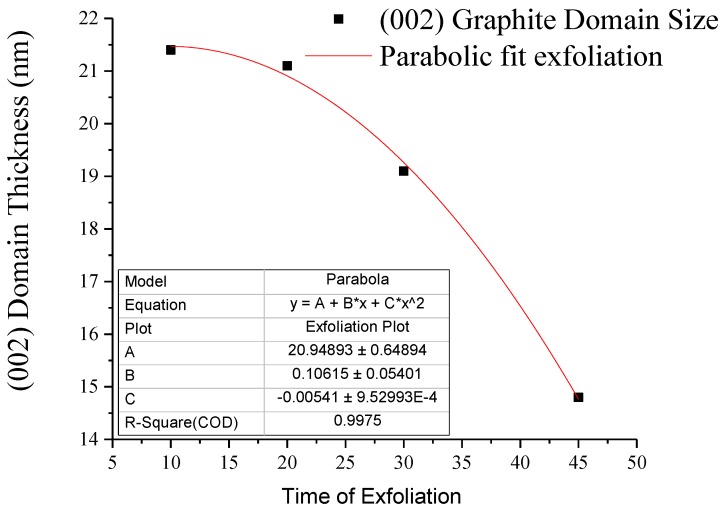
Graphite (002) domain size curve fit.

The starting graphite is analyzed to having a (002) lattice spacing of 0.334 nm, indicative of the stable hexagonal phase [24]. After exfoliated in the presence of elongational flow, the existing hexagonal phase shows an increase in (002) d-spacing, in Table 2. The results from the Figure 2 and Table 2 show a progressive reduction in the average domain size, leading to a 76% reduction in domain size for the graphite portion compared to its native structure. The increase in (002) lattice spacing suggests that the starting hexagonal phase is converting to a turbostratic phase of exfoliated graphite containing composite. The turbostratic form of exfoliate has been known to contain stacks of graphene planes, which have undergone rotations and distortions [25]. Comparing samples G-PA66 30 min and G-PA66 45 min, there is a large difference in both the reduction in (002) domain size and the increase in lattice spacing. This suggests that after 30 min of mixing, a transition in the conversion of the exfoliated graphite and the resulting morphology has occurred.

In Figure 2, a curve fit was constructed to establish a model for exfoliation by the reduction in (002) domain size in graphite. The exfoliation of graphite in PA66 is shown to fit a parabolic model, described as the mechanism for exfoliation. Parabolic models are closely tied to diffusion reaction systems [26,27]. In the diffusion reaction system, the movement of species are modeled as a projecting wave front. In the creation of our graphene reinforced composite, the projecting wave behaves as the diffusing PA66 species between the graphite layers.

The results of the curve fit for the diffusion model shows that the linear equation is second-order with respect to mixing time. Second-order approximations accounts for a linear driving force (LDF) for diffusion a graphite intercalant, from Budzianowski [28]. In his work a second-order model a cyclic adsorption/desorption step change for fluid concentration at the surface was assumed. He found that the half-life for adsorption or distortion was too long and provided no instance for full saturation, while diffusing. In our case, the PA66 diffuses, adsorbs and then desorbs continuously. This process is successive until mixing stops, making it diffusion limited.

(4) $$\left(002\right)\;\mathrm{Domain}\;\left(\mathrm{nm}\right)=20.949+0.106\;t-0.005\;t^{2}$$

**Figure 3:**
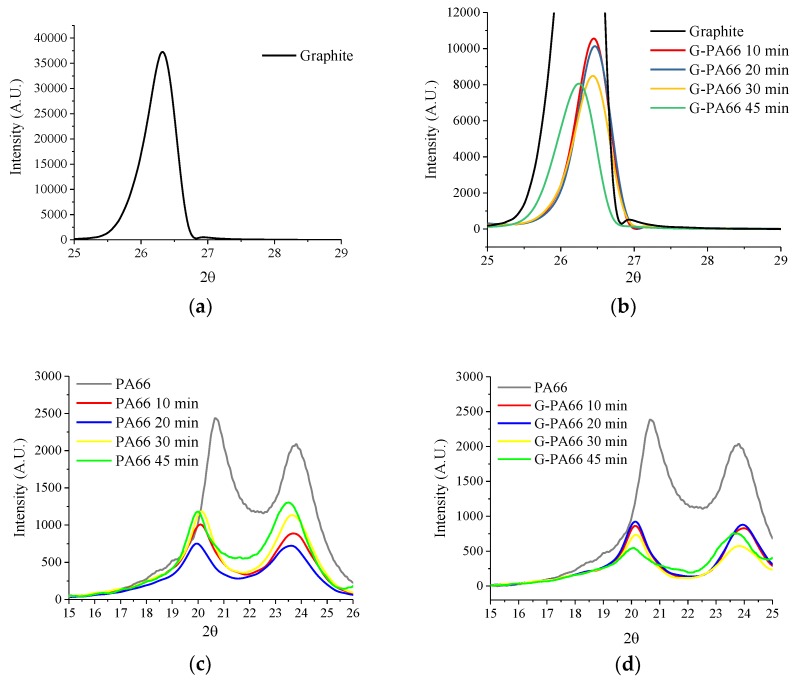
XRD diffraction patterns (**a**) Graphite (**b**) Graphite, G-PA66 10 min, G-PA66 20 min and G-PA66 30 min at Graphite’s (002) c-axis peak-scaled for composite exfoliation (**c**) PA66, PA66 10 min, PA66 20 min and PA66 30 min at the (100) & (010) lattice peaks for PA66 (**d**) PA66, G-PA66 10 min, G-PA66 20 min and G-PA66 30 at the (100) & (010) lattice peaks for PA66.

Diffraction patterns of PA66 under extensional mixing are shown in Figure 3. In PA66, the first crystalline peak at 20.7° 2ϴ, corresponding to the inter-chain hydrogen bonded (100) plane of the amide group for semi-crystalline PA66 [29]. The second crystalline peak for PA66 at 23.6° 2ϴ, for the overlapping diffraction peaks for the (010) and (110) planes, namely the key intra-chain and intersheet periodicities perpendicular to the (100) direction [29]. As mixing time increased for PA66 samples to 30 min, the results do not indicate progressive changes in diffracted domain size or lattice, from Table 2. After 45 min of mixing, (100) domain is drastically reduced by 5.4 nm and (010) by 3.2 nm. The additional mixing time also results in a lattice increase of +0.0038 nm, for the (010) plane. The diffraction data of PA66 suggest that extensive mixing times lead to a reduction in crystal domain and a shift in the (010) plane. The extensive mixing is likely to cause partial degradation which would ultimately results in these crystal morphological changes. 

**Figure 4:**
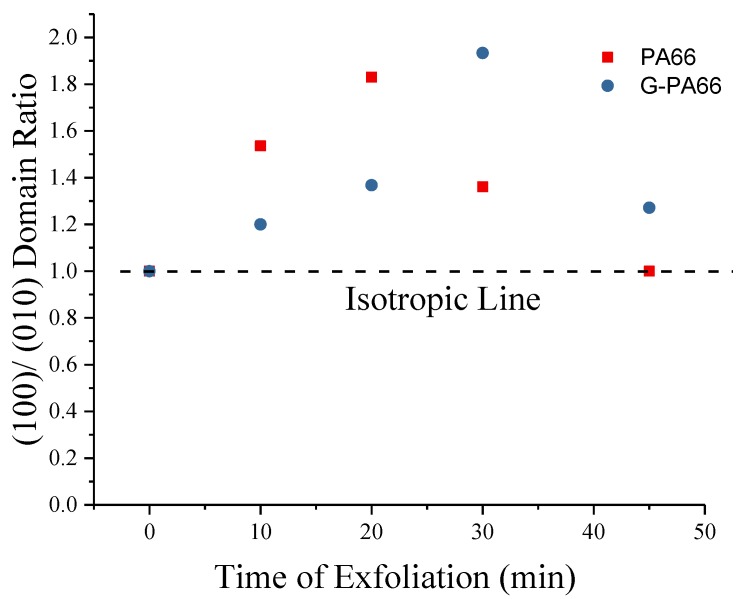
PA66 (100)/(010) domain size isotropy parameter vs. graphite concentration; (010) is (010)/(110).

XRD patterns of PA66, relative to the extensional mixing with graphite, appear in Figure 3d. Relative to Figure 3c, the PA66 diffraction peaks are found to contain close similarities to the graphite exfoliating PA66. After analyzing the diffraction peaks by fitting the resulting lattice and domains, crystal structure information is obtained, Table 2. During graphite exfoliation to 30 min, the crystalline domain of PA66 increases in the hydrogen bonded direction (100) and decreases in the van der Waals direction (010)/(110). The change in domain size suggests a preferential crystallization direction in PA66, likely due to the extensional flow process. In Figure 4, anisotropy of the diffraction peak profiles in pure PA66 versus graphene reinforced PA66 reveals a non-linear dependence on exfoliation time. By the increasing mixing time to 30 min for G-PA66, the inter-sheet (010)/(110) d-spacing increase, Table 2. The non-uniform changes in crystal domain size may allow for preferred crystallization along the surface of the nanoflake and in the hydrogen bonded plane. This suggests preferred direction for PA66 in the (010)/(110) directions and limited growth in the (100) direction.

Extending the time of mixing from 30 min to 45 min with graphite, produces a distinctive change in the G-PA66 lattice structure. G-PA66 shows a lattice increase for both the (100) and (010)/(110) by +0.0030 nm and +0.0037 nm, respectively. This material also changes with a reduction in the hydrogen bonded (100) domain and an increase in the intersheet (010)/(110) domain. The change in the van der Walls (010)/(110) direction trends parallel to the lattice changes in graphite (002). The reduction in (100) d-spacing is likely due to disruptions in the hydrogen bonded network due to graphene and nanographite lattice mismatches, that may increase available surfaces for binding [30].

It is likely that the lattice parameters in the hydrogen bond and the van der Waals direction follows not with the degree at which the graphene is reduced in size but with the (002) lattice spacing between graphene layers beyond 30 min of mixing. The stacking faults in exfoliated graphite invoke changes to the lattice parameters in PA66, which lead to parameter increase. These faults are the results of created turbostratic graphite [31], consisting of an increased and shifted lattice spacing.

### 3.2. Differential Scanning Calorimetry

**Figure 5:**
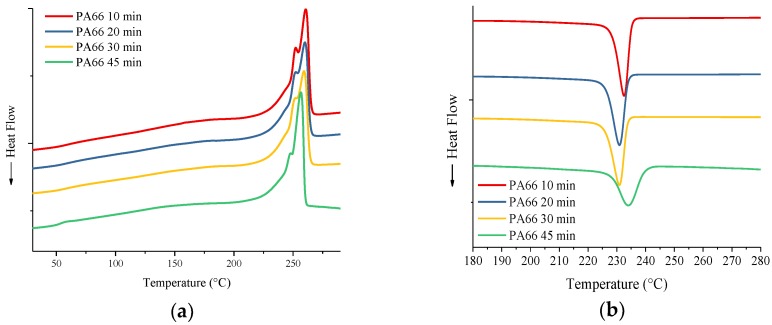

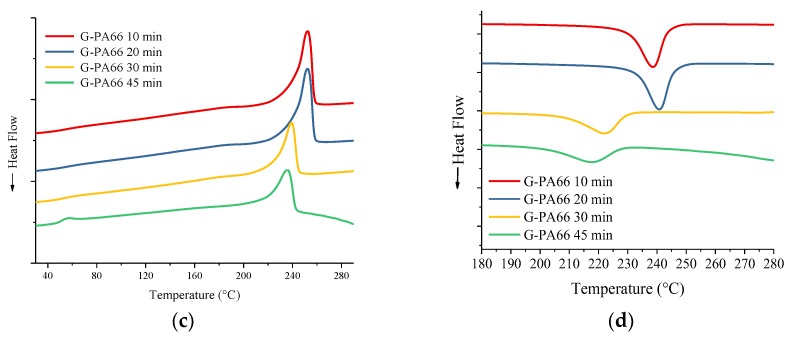
DSC thermograms for PA66 10 min, PA66 20 min and PA66 30 min (**a**) melting and (**b**) crystallization and for G-PA66 10 min; G-PA66 20 min and G-PA66 30 min (**c**) melting (**d**) crystallization.

Thermal properties for PA66 and G-PA66 after 10, 20, 30 and 45 min of elongational mixing appear in Table 3, with corresponding thermograms in Figure 5. Though difficult to notice in Figure 5a,c, the glass transition temperatures for PA66 and G-PA66 were calculated using a baseline tangential method. A line representing the baseline was drawn from 0 °C in the direction of increasing temperature. A deviation from the baseline, at an inflection point in the thermogram, was found to be the glass transition temperature (*T*_g_). Transition temperatures for 10, 20, 30 and 45 min of exfoliation mixing of PA66 & 10, 20, 30, 45 min G-PA66 were found. The glass transitions (*T*_g_) were found to occur at 60, 56, 57, 53, 56, 57, 51, 50 °C respectively. For all the values listed in Table 2. The bulk crystallinity of PA66 was calculated according to (3), for which enthalpy of melting for 100% crystalline PA66 is ΔH_m_ = 197 J/g [32]. The values for ΔH_m_ were adjusted for percent PA66, since G-PA66 samples only have 65 wt % PA66.

(5) $$\mathrm{Weight}\;\%\;\mathrm{Crystallinity}\;=\frac{\Delta H_{m}}{{\Delta H_{m}}_{100}}\times 100$$

In Figure 5a,c, the reheat cycle is shown for PA66 samples, we see the formation of two distinct melting peaks in Figure 5a. Two melting peaks are due to a bimodal distribution of crystallites formed by an intermediate heating and cooling rate of 10 °C/min [33]. Since the rate chosen is constant, the thermal characteristics of the crystallites are dependent on the time of mixing and crystal structure formed. As the mixing time increases, the first melting peak reduces in intensity and the secondary melting peak broadens. This broadening of the overall melting in PA66 is indicated by the reduction in onset melting temperature relative to *T*_m_. As the mixing time increases, *T*_g_, *T*_m_, *T*_c_ and ΔH_c_ all decrease. However, there is no consistent trend with ΔH_m_ results in Table 3 which produces a small increase in % crystallinity as mixing continues beyond 20 min.

In Figure 5c, the reheat scans of the G-PA66 samples show a single melt peak; suggesting a narrow distribution of crystallites formed, compared to the bimodal curve in neat PA66. G-PA66 produces an increased crystallization temperature (*T*_c_) and percent crystallinity for 10 and 20 min of mixing; symptomatic to heterogeneous nucleation of PA66 in graphene/nanographite presence. As the exfoliation time continues beyond 30 min, there a reduction in *T*_g_, *T*_m_ and *T*_c_. These properties are much lower than all the other materials, suggesting a drastic change occurs at 30 min and beyond, in its structure.

Compared to pristine PA66 at 30 min, G-PA66 at the same exfoliating time, has nearly equivalent values of percent crystallinity but has a much lower *T*_m_ and *T*_c_. The reduced crystallization previously mentioned, suggests the suppression of crystallization to occur. The exfoliated graphitic nanoflake in the matrix is likely to cause a subdued rate of crystallization [34,35]. This suppression lead to a reduced crystallization temperature *T*_c_ of 9 °C. The same results appear for the *T*_g_, with 45 min of exfoliation resulting to a 6 °C reduction than the lowest *T*_g_. The crystallization suppression is shown sensitive to the percent crystallinity calculated, which suggest lower *T*_c_ depression leads to lower crystallinity.

Identifying how the crystal structure relates to the thermal stability of G-PA66 composite, we see that the hydrogen bonded lattice parameters play an important role. This is not represented in the neat PA66, since the differences between 20 and 30 min are nominal. G-PA66 mixed at 20 min; having the least modified hydrogen bonded lattice, has relatively higher thermal properties and percent crystallinity. The thermal property variations occurring through reduction of the hydrogen bonded lattice, suggesting there is a relation to the structure in the PA66 of the composite. From the increased crystallization temperature Tc and percent crystallinity, we know this is evidence of surface crystallization occurring in the G-PMC (10 & 20 min). The cause of crystallization temperature depression in G-PA66 mixed at 30 min might be explained by the exfoliated graphene acting as unstable crystals growth surfaces. These surfaces form disrupted hydrogen bonded sites; imparting themselves between the formed crystals, suppressing their crystallinity and thermal stability [36].

### 3.3. Scanning Electron Microscopy

**Figure 6:**
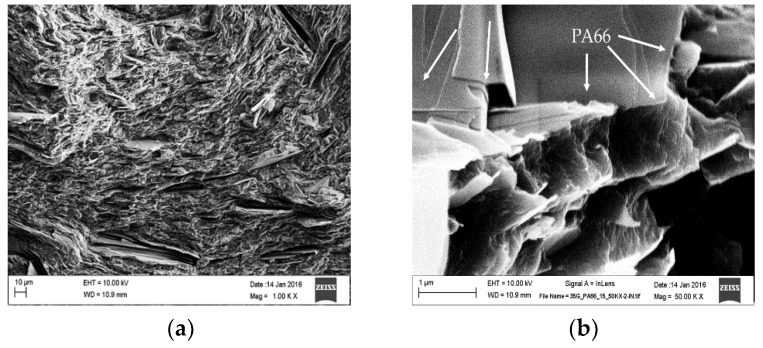
(**a**) Surface of cryogenically fractured G-PA66 20 min composite at low magnification; (**b**) Graphitic edge overhanging polymer in G-PA66 20 min composite.

In all stages of the exfoliation process, it is important to note that there exist both nanographite and many layer graphene flakes, even though the average graphite domain is calculated to be 14.8 nm. This is suggested, due to the fact that few to many layer graphene crystallites in the (002) direction are weakly diffracted. The weakly diffracted domains make the crystals unresolved from the background. That being said, the first thing to notice is the lack of large flakes of graphite present on the fractured surface. The lack of visibly large flakes suggest that nearly all the graphite were reduced in thickness but retained relatively large surface area in Figure 6. The reduction in graphite flake thickness provides improved dispersion of nanographite and many layer graphene sheets, making it difficult in locating the surfaces of the PA66 matrix. When cryogenically fracturing the extrudate in Figure 6a, voids are left behind on the surface of the samples. These voids are holes left by the sliding of graphitic sheets not interacting with the matrix, as they are being pulled out from their center. The nature of the pullout structure left behind suggests some flakes are ‘locked in.’ It is also interesting to note that pulled out sections leave behind corrugated steps at the edges of exfoliate, seen in Figure 6.

When looking at Figure 6b, a more detailed image is shown of the step morphology left by the process. Following along the steps in Figure 6b we notice what appears to be a rolled edge, with the direction of rolling applied to the left in the micrograph. The development of this rolled edge structure seen in the image, is presumably caused by frictional forces imposed by the matrix. The peeling leaves behind a nanosheet, which is shown to be ripped at the edge. If you follow the edge where the ripping occurs, you can see PA66 attached or adhered to its surface. This rolled structure with the edged attached PA66 is an indication of matrix interactions with the nanoflake graphitic structure. The morphology provides clues to the mechanism of exfoliation in this process.

**Figure 7:**
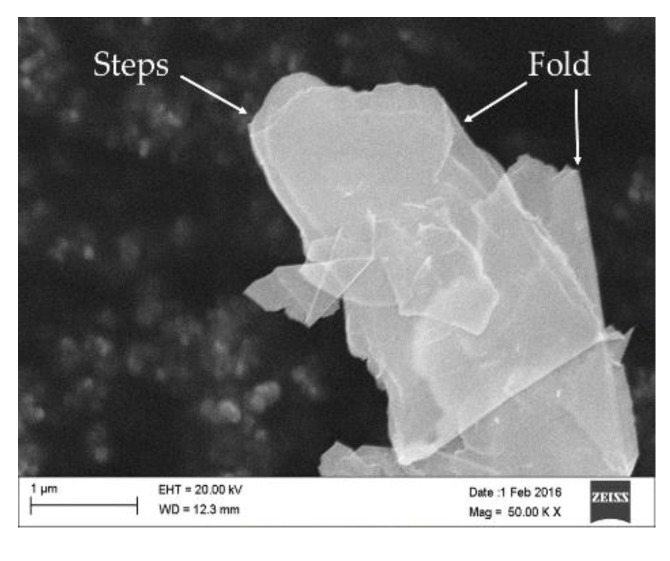
Cryogenically milled G-PA66 20 min showing transparency of exfoliated graphene flake, at high magnification.

To observe the created graphene in the absence of the extrudate matrix, cryogenic milling was performed on the 30 min G-PA66. In Figure 7, large area nanographite and many layer graphene is shown having what looks like an elongated shape. Focusing on the edges of the newly created flakes, a jagged and then smooth structure is noticed. By following those edges on the right side of the micrograph, the rolled section appears smooth. Comparing this discovery to the previous Figures, this suggests that the other smooth sections are folded over sheets of many layer graphene, created by the frictional force of the molten polymer. 

What is interesting in Figure 7, is the transparency of the large area flakes to the electron microscope. Folded sections, as previously mentioned, shows the presence of additional wrinkles and folding structures under the layered graphene sheets. The transparency suggests that graphene is being created, otherwise transmission would be extremely difficult for nanographite flakes. 

What is difficult to find, is a distinct indication of the PA66 with the graphene in Figure 6b. What we do find is that the transparent surfaces of the flake are littered with bright surface and edge features. What these features represent is still not fully known. We do know that given the analysis technique, Insulators/nonconductors in SEM cause charge buildup in their surface. In that this charge buildup presents itself as bright spots in produced micrographs [37]. PA66 is known to have such low conductivity, that it is an electrical insulator. The bright spots are likely to be edges containing PA66, PA66 alone, or a combination of both. Observing the micrographs, we see the pristine surfaces, which stress the point that our novel exfoliation preserves the in-plane surface structure of graphene. A traditional chemical method of graphene creates surface defects, wrinkles and holes; which never simply retain such pristine surfaces [38,39].

### 3.4. Transmission Electron Microscopy

**Figure 8:**
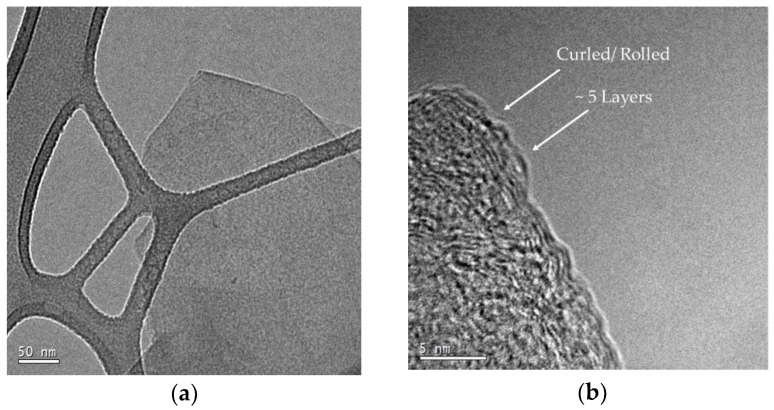

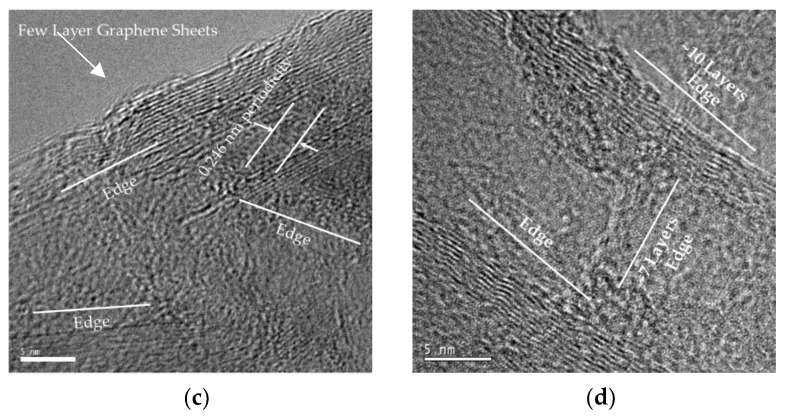
(**a**) Low Magnification micrograph of isolated 20 min G-PA66 flake on lacey carbon surface; (**b**) High magnification micrograph of rolled edge of many layer graphene; (**c**) High magnification micrograph of edge containing nanographite, many layer and few layer graphene; (**d**) High magnification micrograph containing many layer graphene.

The first thing noticed from the HR-TEM analysis is that from the low magnification image, the process produces graphene and nanographite composite flakes of varying sizes Figure 8a. Theses sizes consist of a combination of few layer graphene (2–5 layers), many layer graphene (5–10 layers) and the rest containing nanographite (<100 nm in thickness) [40]. In Figure 8b, 5 individual layers of graphene sheets are identified, while having the on the edge structure in focus. Following along the same figure, the sheets are noted to folding upward; representative of a feature inherent to graphene, due to stresses at the edge [41]. Looking closer in Figure 8c, a continuous structure is noticed on the steps of the nanographite (the number of counted steps exceeds 10 layers), containing a smaller periodicity on the order of ~0.246 nm in size. 0.246 nm is known to be the lattice parameter of graphite, representing the distance between carbon atoms along a single direction in the surface of a graphene flake. Given the size of the smaller periodicity, the surface structure is believed to be a 1 to 2 layer graphene sheet draped along the edge. This is an indication from the previous statement that nanographite to graphene with a (002) smaller than 14.8 nm exist within the G-PA66 composite. This provides evidence that a multiscale layers of graphene and nanographite exists within this composite.

Taking a step back and looking at the arrangement of the flakes, many layer graphene and nanographite are shown to be orientated in multiple directions in Figure 8c,d. These structural disruptions are indicative of turbostratic graphite; where the c-axis stacking sequence is either rotated or completely disrupted. These turbostratic layers are confirmed by the diffraction results; where they exhibit an increase in the c-axis spacing of the graphene layers. In this case, G-PA66 that was characterized, was mixed for 45 min; which suggests this to have a spacing of 0.3386 nm; having a 0.0042 nm lattice increase.

## 4. Conclusions

In our novel process, elongational exfoliation in the molten phase led to the formation of pristine, large area nanographite and multilayer graphene flakes. These results are confirmed from HR-TEM and morphology in electron microscopy. From HR-TEM and XRD, the resulting process identified that the graphite was exfoliated, leading to the disruption of the graphene layers in the composite and a 76% reduction in the initial (002) domain size. In-situ, a misalignment is described by a rotation of the graphene layers and conversion to a turbostratic phase. The model of graphite exfoliation revealed that the process is diffusion driven, with intercalation of the PA66 within the graphite galleries. In addition to diffusion, the process is found to tear and fold the outer layer sheets in graphite creating instance for the formation of dangling bonds. The results of which, preclude to a solvent-less method to functionalization graphene. The morphology created changes in the hydrogen bonded structure, given by changes in the lattice and domain in the (100) and (010) crystalline plane. Changes lead to preferential crystal growth in the hydrogen bonded direction, resulting in changes to the *T*_m_ and *T*_g_. Due to these changes, the graphene exfoliate is found suppresses the crystallization in PA66, leading to a modified microstructure. 

The G-PMC created by the process, shows graphene to be created due high shear. The results show a single step approach to create as well as modify graphene. Further investigation into this work may reveal a better description of the interfacial properties of these two species and the type of bonding present. This may provide ways of modifying the interface functionality in a scalable process and further exploiting the capabilities of elongational flow. This in-turn drives down cost, which is an important variable in adaptation and scalability.

## Figures and Tables

**Table 1:** Processing Matrix.

Mixing time (min)	Control materials	Composite materials
10	PA66	G-PA66
20	PA66	G-PA66
30	PA66	G-PA66
45	PA66	G-PA66

**Table 2:** Diffraction data for Graphite, PA66 and G-PA66.

Materials	Mixing time (min)	PA66 d (100) (nm)	PA66 (100) Domain (nm)	PA66 d (010)/(110) (nm)	PA66 (010)/(110) Domain (nm)	Graphite d (002) (nm)	Graphite (002) Domain (nm)
Graphite	-	-	-	-	-	0.3344	62.7 (4.1)
PA66	-	0.4407	4.4 (0.2)	0.3878	4.4 (0.7)	-	-
PA66	10	0.4427	8.6 (0.4)	0.3753	5.6 (0.2)	-	-
PA66	20	0.4453	9.7 (0.6)	0.3768	5.3 (0.2)	-	-
PA66	30	0.4414	8.3 (0.4)	0.3758	6.1 (0.2)	-	-
PA66	45	0.4435	2.9 (0.9)	0.3796	2.9 (0.5)	-	-
G-PA66	10	0.4404	8.4 (0.4)	0.3719	7.0 (0.2)	0.3360	21.4 (0.3)
G-PA66	20	0.4405	9.3 (0.5)	0.3720	6.8 (0.1)	0.3358	21.1 (0.2)
G-PA66	30	0.4393	11.6 (0.8)	0.3735	6.0 (0.1)	0.3363	19.1 (0.2)
G-PA66	45	0.4423	8.9 (1.7)	0.3772	7.0 (0.3)	0.3386	14.8 (0.1)

**Table 3:** Differential scanning calorimetry results for PA66 and G-PA66.

Material	Mixing time (min)	*T*_g_ (°C)	Onset melting (°C)	*T*_m_ (°C)	ΔH_m_ (J/g)	Onset crystallization (°C)	*T*_c_ (°C)	ΔH_c_ (J/g)	Crystallinity %
PA66	10	60	246	261	72	235	233	70	~36
PA66	20	56	244	260	69	233	231	68	~35
PA66	30	57	243	259	71	233	231	66	~36
PA66	45	53	245	256	73	238	234	59	~37
G-PA66	10	56	241	252	97	243	239	85	~49
G-PA66	20	57	243	254	103	245	241	94	~52
G-PA66	30	51	226	239	69	229	222	74	~35
G-PA66	45	50	218	236	48	227	217	38	~24
